# Micro-epidemiology of malaria in an elimination setting in Central Vietnam

**DOI:** 10.1186/s12936-018-2262-0

**Published:** 2018-03-19

**Authors:** Melanie Bannister-Tyrrell, Nguyen Xuan Xa, Johanna Helena Kattenberg, Nguyen Van Van, Vu Khac Anh Dung, Truong Minh Hieu, Nguyen Van Hong, Eduard Rovira-Vallbona, Nguyen Thanh Thao, Tran Thanh Duong, Anna Rosanas-Urgell, Koen Peeters Grietens, Annette Erhart

**Affiliations:** 1 Department of Public Health, Institute of Tropical Medicine, Antwerp, Belgium; 2 National Institute of Malariology, Parasitology and Entomology, Hanoi, Vietnam; 3 Department of Biomedical Sciences, Institute of Tropical Medicine, Antwerp, Belgium; 4 Quang Nam Provincial Center for Malaria Control, Tam Ky, Vietnam; 5 Medical Research Council Unit, Fajara, The Gambia

**Keywords:** Micro-epidemiology, Mixed methods, Malaria elimination, Residual transmission, Vietnam

## Abstract

**Background:**

In Vietnam, malaria persists in remote forested regions where infections are spatially heterogeneous, mostly asymptomatic and with low parasite density. Previous studies in Vietnam have investigated broad behavioural concepts such as ‘engaging in forest activities’ as risk factors for malaria infection, which may not explain heterogeneity in malaria risk, especially in malaria elimination settings.

**Methods:**

A mixed methods study combining ethnographic research and a cross-sectional survey was embedded in a 1-year malariometric cohort study in three ethnic minority villages in South Tra My district, Quang Nam Province in Central Vietnam. Qualitative data collection included in-depth interviews, informal conversations and participant observations over a 2-month period, and the findings were used to develop the questionnaire used in the cross-sectional survey. The latter collected data on evening activities, mobility patterns and household characteristics. The primary outcome, recent exposure to malaria, was defined using the classification and regression tree method to determine significant changes in antibody titres during the year preceding the survey. Risk factor analyses for recent exposure to malaria were conducted using logistic regression.

**Results:**

22 in-depth interviews and numerous participant observations were recorded during the ethnographic research (April to June 2015), and 160 adults (86% response rate) responded to the cross-sectional survey (November to December 2015). Recent exposure to *Plasmodium falciparum* malaria was estimated at 22.9 and at 17.1% for *Plasmodium vivax*. Ongoing malaria transmission appears to be maintained by activities that delay or disrupt sleeping in a permanent structure in which a bed net could be hung, including evening drinking gatherings, fishing, logging in the forest and outdoor TV watching.

**Conclusions:**

Vector control tools for outdoor evening activities in villages as well as at farms, forest and river locations should be incorporated into current malaria elimination efforts in Central Vietnam. Micro-epidemiology studies using mixed-methods designs can provide a comprehensive understanding of the malaria risk at fine spatial scales and better inform the implementation of targeted interventions for malaria elimination.

**Electronic supplementary material:**

The online version of this article (10.1186/s12936-018-2262-0) contains supplementary material, which is available to authorized users.

## Background

The burden of malaria in the Greater Mekong Subregion (GMS) has declined substantially over recent decades [1]. The Vietnamese government is aiming to achieve national malaria elimination by 2030 in accordance with the GMS regional malaria elimination strategy [1], buoyed by the success of an intensive malaria control programme that led to a more than 90% reduction in malaria cases since 2000 [2]. In Vietnam, malaria persists at low prevalence mainly in remote forested regions, many of which are inhabited by ethnic minority populations practicing subsistence slash and burn agriculture [3–7]. Within these persisting transmission foci, malaria is characterized by a high prevalence of asymptomatic infections with considerable fine-scale spatial heterogeneity, whereby malaria risk can vary substantially within and between villages [5, 8]. Determinants of fine-scale heterogeneity in malaria infection in low transmission settings are in general not well understood [9–11]. In settings approaching elimination, characterizing risk factors amongst sub-groups who continue to be at risk of malaria, despite overall declining incidence and implementation of malaria control measures, is crucial, as this small proportion of the population, often with asymptomatic infections, may serve as a reservoir of infections for whenever local conditions are permissive for malaria transmission [12, 13].

Studies from Central Vietnam have previously shown that age, bed net use and spending nights at farms and fields located in forested areas away from the village were important risk factors for malaria infection [4–6]. However, these risk factors were identified in studies that were conducted prior to the upscaling of LLIN distribution and awareness campaigns from 2010 onwards, which included promotion of LLIN use at forest farms as well as in villages. No recent studies have investigated socio-behavioural risk factors for malaria infection in Central Vietnam, which may have shifted since the onset of expanded malaria control activities. Furthermore, the primary vector in Central Vietnam, i.e. *Anopheles dirus* senso stricto demonstrates a preference for outdoor and early evening biting [14, 15], which implies that outdoor early evening activities may favour exposure to biting vectors that cannot be prevented by sleeping under LLINs at night, thus some risk factors relating to evening outdoor exposure may have been missed in previous studies.

Therefore, this study aimed to gain a detailed understanding of human behaviours during vector biting times that may increase the risk of malaria infection in a low-transmission setting, to inform further improvement of malaria elimination activities in this region.

## Methods

### Study setting and population

The study was based in Tra Cang Commune, in Nam Tra My district, Quang Nam Province, Central Vietnam. Tra Cang had a population of approximately 4000 people at the time of the study, almost all Xe Dang, an ethnic minority population mostly living in the central mountainous inland regions of Vietnam. Tra Cang commune comprises seven administratively-defined villages each comprising several hamlets of Xe Dang households. Most Xe Dang families also maintain a house or hut at their farms and rice fields, and reside there according to seasonal work requirements.

Both *Plasmodium falciparum* and *Plasmodium vivax* malaria transmission can occur year-round, with two peaks of transmission in June/July and October/November. Since 2005, malaria cases had declined substantially in Tra Cang, but a local outbreak occurred in 2012 and 2013, followed by a steady decrease since 2014 (Kattenberg et al., pers. comm.). Health care services in Tra Cang commune includes village health workers (one to two per village), a commune health centre (CHC) staffed by Xe Dang and Kinh (majority Vietnamese ethnic group) staff, and the district hospital, which is about half an hour by motorbike from the CHC. Malaria control activities include distribution of long-lasting insecticidal nets (LLINs), as well as provision of malaria testing (rapid diagnostic test and microscopy) and treatment (dihydroartemisinin-piperaquine and chloroquine were first-line treatment for *P. falciparum* and *P. vivax,* respectively, at the time of the study) at the CHC and at two village malaria posts in Village 5 and Village 7. Indoor residual spraying of houses had not been systematically undertaken at the time of the study, though public buildings such as schools were sprayed during the last outbreak in 2012/13. Intensive treatment-based control efforts were conducted between 2012 and 2014 in response to the outbreak, including treating entire households with the first-line treatment where one malaria-positive case was detected by rapid diagnostic test (RDT), and treating entire hamlets when RDT-positive cases were detected in two or more households (A. Erhart, pers. comm.).

### Study design

A sequential mixed methods study [16], comprising a qualitative ethnographic strand (April to June 2015) followed by a cross-sectional survey (November to December 2015) was conducted ancillary to a cohort study (‘MAPARES study’), that followed the entire population of three hamlets (Tu Nak and Tak Lang in Village 5, Xe Xua in Village 7) through six consecutive malariometric surveys and passive case detection (PCD) between November 2014 and December 2015. In the cohort study, finger prick blood samples were collected and stored for light microscopy and PCR detection of *Plasmodium* spp. infection in all six surveys, as well as for serological analysis of antibody levels to two recombinant *P. falciparum* antigens (AMA1 [17] and GLURP-R2 [18]), and two recombinant *P. vivax* antigens (AMA1 [19] and MSP_1–19_ [20]) from the first and last survey. By the end of the cohort study, four asymptomatic infections had been confirmed by PCR while no cases were detected by PCD.

In the mixed methods study, the qualitative ethnographic strand aimed to explore the local context and identify possible risk factors for malaria that had not previously been identified, and the cross-sectional survey aimed to test whether these risk factors were associated with recent malaria infection.

### Data collection and analysis

Data collection and analysis for the qualitative and quantitative strands are described separately below, and reported according to the STROBE statement for reporting observational epidemiological research [21], supplemented by COREQ guidelines for reporting qualitative research [22].

### Qualitative strand

#### Research team

The field research team comprised the first author, the local principal investigator (NXX) and research assistants from the National Institute of Malariology, Parasitology and Entomology (NIMPE), Hanoi, previously trained in qualitative ethnographic research methods. The study team resided in Tra Cang for 2 months (April to June 2015) supported by the health staff at the Tra Cang CHC. The research team from NIMPE had extensive field experience in similar settings, and had previously visited the study setting on several occasions during the MAPARES study.

#### Participants and sampling

An open-ended research design centred on ethnographic methods guided the fieldwork. A purposive and adaptive sampling strategy was used to select informants, based on criteria such as history of malaria infection, mobility patterns, age, sex, and occupation. Emerging findings led to refinement of the sampling strategy and topic guides in order to maximize both variation and depth of information in the sample.

#### Data collection

Data collection methods included audio-recorded in-depth interviews, informal conversations (not audio-recorded but recalled and summarized in field notes), informal group discussions, and participant observation. Participants were usually first visited at their hamlet houses, but the study team also accompanied participants to their farms and fields, especially those who slept overnight in their plot huts. Interviews were also conducted in the general store, schools and CHC located in the administrative centre of Village 5. All interviews were conducted in Vietnamese, with local Xe Dang guides assisting as translators for some interviews. Field diaries and memos were maintained to aid data interpretation.

#### Data analysis

Data were intermittently analysed in the field concurrent with data collection, leading to refinement of topic guides and further sampling. At the end of the fieldwork period, all data were imported into NVivo (NVivo for Mac v11.40, QSR International Pty Ltd) for data management. Descriptive open coding and analysis focused on agricultural and social activities occurring at times and locations that may be associated with exposure to local vectors’ bites, but also describing the general socio-cultural context, health-seeking behaviour and local understandings of malaria.

### Quantitative strand

#### Participants

A simple random sample of 186 individuals aged 16 years and over was drawn from the population census of two hamlets (Xe Xua and Tu Nak), where malaria infections had been detected during the MAPARES study. The sample size calculation was based on detecting, with 80% power and at 5% significance, a minimum 20% difference in the odds of recent malaria exposure (defined by serology), for individuals staying overnight at forest farms and fields, compared to individuals who do not. As malaria sero-prevalence was unknown at the time of the cross-sectional survey, the maximum sample size (n = 186 at 50% exposed-group prevalence) was used.

#### Data collection

The survey comprised a close-ended structured paper-based questionnaire. The questionnaire was piloted and revised, and the final version back-translated into English and checked for clarity. Trained surveyors from NIMPE administered the questionnaire at respondents’ households. Surveyors made up to three attempts to reach selected individuals. Data were double-entered and cleaned using Epi Info (Center for Disease Control and Prevention, Atlanta, USA).

#### Variables

The primary outcome was defined as recent exposure to malaria infection, estimated by serology and defined below. Serology was used because only four PCR-positive cases were detected during the six surveys, but also because serology outcomes may be more robust markers of exposure to malaria infection since it reflects cumulative changes in antibody titres over time, while PCR only detects malaria infections present at the time samples were taken [23–28]. Individual mean percent-positive antibody levels (mean ‘PP’; mean value among duplicates of the sample optical density (OD) measured as a proportion of the strong positive control OD) against two *P. falciparum* antigens (AMA1, GLURP2) and two *P. vivax* antigens (AMA1, MSP1) were defined for all participants in the first and last (sixth) MAPARES surveys. Subsequently, these continuous variables were categorized into four groups indicating the extent of seropositivity (strong positive, weak positive, grey (transition) zone, negative) using the classification and regression tree (CART) method [29] and implemented using CART ^®^ software (Salford Systems, San Diego, USA). Cut-off points were defined using CART regression on each antibody distribution in the first survey, and applied to the sixth survey (Additional file 1). Regression trees were also computed for the changes in mean PPs from Survey 1 to Survey 6, to identify important minimum absolute increase and relative percent decrease that could be considered indicative of recent exposure and non-exposure, respectively. The following algorithm was used to define ‘recent exposure to malaria infection’:

- Individual was negative or grey zone in Survey 1, and moved to a higher category (weak or strong positive) with a minimum absolute increase in PP in Survey 6, for one or more antibodies;
- Individual was weak or strong positive in Survey 1, and had either a minimum absolute increase in PP in Survey 6, or less than the relative minimum decrease, for one or more antibodies;
- Individual was strong positive in Survey 6 for one or more antibodies, if missing from Survey 1;
- Individual was strong positive in Survey 1 for one or more antibodies, if missing from Survey 6.

#### Exposures

**Fig. 1:**
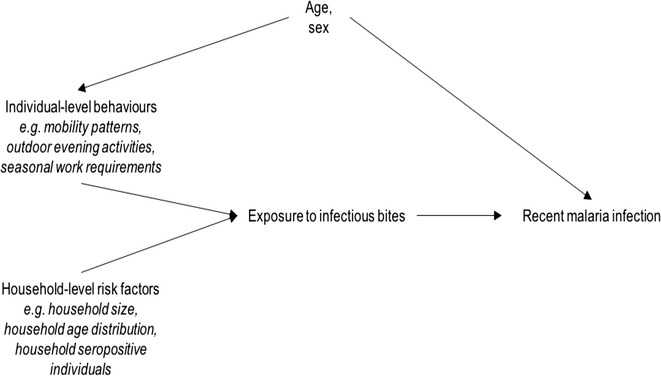
Causal diagram for association between individual and household-level risk factors for exposure to recent malaria infection

The research focused on identifying activities that may increase exposure to vector bites as risk factors for recent exposure to malaria infection. Based on the qualitative strand, the questionnaire included items about time spent in locations of presumed differential exposure to biting vectors (e.g. deep forest, forest farms and fields, river, hamlet), as well as evening and night time activities in these different locations that delay usual sleeping time and/or disrupt bed net use (e.g. drinking, outdoor TV watching, hunting, fishing). Several household-level variables were also included, such as household size [30], number of children, household ITN/LLIN coverage, housing construction, and presence of another recently exposed individual. The assumed causal relationships between these variables is depicted as a directed acyclic graph [31] in Fig. 1, and formed the basis of the modelling strategy.

#### Statistical analysis

Logistic regression models with robust standard errors to account for clustering by household were used to calculate crude and adjusted odds ratios for the association between exposure variables and recent malaria exposure, separately for *P. falciparum* and *P. vivax*. In line with the causal modelling approach, the emphasis was on appropriate adjustment for confounding rather than statistical significance alone, with the number of exposure variables balanced against data scarcity, which was assessed by inflation of standard errors upon inclusion of additional variables. All exposure variables with p values less than 0.2 in the unadjusted analyses were included in the multi-variable models. Given the small sample size relative to the number of exposure variables, individual-level variables with p value exceeding 0.2 were removed from the multi-variable models, before household-level variables were included. Household-level variables were then retained if their inclusion meaningfully shifted the effect estimates of exposure variables without substantially increasing standard errors. Age and sex were forced (confounding) variables in all models. Sub-group analyses were conducted for respondents who slept overnight at their forest farms and fields, as their overall exposure profile was considered to differ from respondents who always slept in their hamlet. Sensitivity analyses restricted to individuals with serological data in both Survey 1 and Survey 6 were run. All regression analyses were conducted in Stata/IC 13 (StataCorp LLC, College Station, Texas, USA).

### Ethics

The study was approved by the institutional review boards of the Institute of Tropical Medicine, Antwerp, Belgium (ITM 1043/15), and the National Institute of Malariology, Parasitology and Entomology, Hanoi, Vietnam. Participants were included in the study if they were aged 16 years and over (the age of majority in Vietnam) and verbal consent was given. Ethical review and consent procedures were additional to those in the MAPARES study, which had separate approvals (ITM-UZA 958/14).

## Results

### Characteristics of study population

In the qualitative strand, 22 in-depth interviews were transcribed and translated, 10 informal conversations and numerous observations were recorded. Informants included health care workers, community leaders and officials, farmers, loggers and secondary school students. In the quantitative strand, 160 (86%) participated in the survey. All survey respondents were of Xe Dang ethnicity, 52% were males and 48% were females, with a mean age of 35 years. Most (92%) were farmers, and 62.5% had not been educated beyond primary school level (Table 1).

**Table 1:** Characteristics of cross-sectional survey study population (quantitative strand)

Variable	Category	n	(%)
Demographic variables		x̅	(sd)
Age		34.77	(14.92)
		n	(%)
Sex	Male	83	(51.88)
	Female	77	(48.12)
Ethnicity	Xe Dang	160	(100)
Occupation	Farmer	147	(91.88)
	Other	13	(8.12)
Education level	No schooling	53	(33.12)
	Primary school	47	(29.38)
	Secondary school	50	(31.25)
	High school	10	(6.25)
Hamlet variables			
Drinks in evening in hamlet	Daily	19	(11.95)
	Less than daily	140	(88.05)
Evening TV watching in hamlet	Does not watch TV	90	(56.25)
	Watches TV at home	28	(17.5)
	Watches TV in another house	42	(26.25)
Uses bed net in hamlet house	Never	4	(2.56)
	Sometimes	35	(22.44)
	Always	117	(75)
Condition of bed net in hamlet	No bed net	4	(2.5)
	Bed net with holes	60	(37.5)
	Bed net without holes	96	(60)
Hamlet household variables		x̅	(sd)
Number of children in household		3.08	(1.81)
		n	(%)
Household bed net ratio	1–2 people per net	46	(29.68)
	> 2 people per net	109	(70.32)
Other seropositive household member	No	77	(55)
	Yes	63	(45)
Hamlet house structure	On ground	24	(15.2)
	On stilts	134	(84.8)
Farm variables			
Stays overnight at farm	No	88	(55)
	Yes	72	(45)
Amongst farm sleepers:			
Duration of stay at farm	Stays up to 1 week	56	(35)
	Stays more than 1 week	16	(10)
Drinks in evening at farm	Yes	28	(17.5)
	No	44	(27.5)
Uses bed net at farm	Yes	65	(40.6)
	No	7	(4.4)
Farm bed net condition	No bed net	7	(4.38)
	Bed net with holes	37	(23.12)
	Bed net without holes	28	(17.5)
Forest and river variables			
Goes fishing in the evening	Yes	20	(12.5)
	No	140	(87.5)
Goes to forest in the evening	Yes	74	(46.25)
	No	86	(53.75)
Evening forest activities	Logging	6	(3.75)
	Collect firewood	18	(11.25)
	Hunting	52	(32.5)
	Collect forest food	38	(23.75)
Other variables			
MAPARES cohort participation	Partial (1 to 5 screenings)	105	(65.62)
	Full (6 screenings)	55	(34.38)

Serological data was available for 140 of the 160 cross-sectional survey participants, including 125 who participated in the first screening, 119 for the sixth screening, and 104 for both screenings. The proportion of respondents categorized as weak or strong positive declined overall between Survey 1 and Survey 6 for all antibodies (Additional file 2). There were 32 (22.9%) respondents whose *P. falciparum* antibody levels remained stable or increased between the two surveys, and 24 (17.1%) respondents with stable or increasing *P. vivax* antibody levels who met the criteria for ‘recent exposure to malaria infection’.

### Risk factors for recent exposure to malaria infection

#### Hamlet-based activities

Access to a bed net in hamlet houses was reported to be very high (97.5%), and 75% reported always sleeping under a bed net (Table 1), however only 30% of households had a household bed net ratio of 1–2 people per bed net. Only 10 (6.25%) surveyed individuals reported sleeping in another house in the same hamlet, 8 of whom reported using bed nets when sleeping in a different house. Overall, neither individual bed net use when sleeping in the hamlet or bed net condition was associated with recent *P. falciparum* or *P. vivax* infection (Tables 2, 3).

**Table 2:** Odds ratios for association between exposure variables and recent *Plasmodium falciparum* exposure by serology, adjusted for age and sex only

Variable	n recently exposed (%)		OR	(95% CI)	p
Age	44.44	(15.14)	1.05	(1.02–1.08)	< 0.001
Sex					0.06
Male	11	(16.67)	1		
Female	21	(28.38)	2.36	(0.98–5.70)	
Drinks in hamlet in evening					0.93
No	12	(23.08)	1		
Yes	20	(22.73)	1.05	(0.41–2.69)	
Frequency of evening drinking in hamlet					0.015
Daily	8	(42.11)	4.05	(1.31–12.53)	
Less than daily	24	(20)	1		
Evening TV watching					0.18
Does not watch TV	24	(28.24)	1		
Watches TV at home	4	(16.67)	0.39	(0.1–1.43)	
Watches TV in another house	4	(12.9)	0.43	(0.13–1.43)	
Uses bed net in hamlet house					0.79
Never	2	(50)	1		
Sometimes	6	(21.43)	0.64	(0.05–7.49)	
Always	23	(22.12)	0.51	(0.05–5.1)	
Condition of bed net in hamlet house					0.75
No bed net	1	(25)	1		
Bed net with holes	15	(27.78)	1.29	(0.11–14.6)	
Bed net without holes	16	(19.51)	0.92	(0.08–10.23)	
Number of children in household	2.38	(1.9)	0.81	(0.64–1.03)	0.09
Household bed net ratio					0.06
1–2 people per net	14	(33.33)	1		
> 2 people per net	17	(18.28)	0.42	(0.17–1.03)	
Other *P. falciparum* seropositive household member					0.01
No	22	(8.43)	1		
Yes	20	(15.38)	3.18	(1.30–7.78)	
Hamlet house structure					0.11
On ground	7	(33.33)	1		
On stilts	25	(21.37)	0.42	(0.14–1.22)	
Stays overnight at farm					0.22
No	15	(19.74)	1		
Yes	17	(26.56)	1.71	(0.72–4.05)	
Duration of stay at farm					0.06
Does not stay	15	(19.74)	1		
Stays up to 1 week	11	(22)	1.24	(0.48–3.19)	
Stays more than 1 week	6	(42.86)	5.07	(1.33–19.23)	
Drinks in evening at farm					
Does not stay at farms	15	(19.74)	1		0.44
Yes	7	(29.17)	1.95	(0.63–6.00)	
No	10	(25)	1.58	(0.60–4.21)	
Uses net at farm					0.41
Does not stay at farms	15	(19.74)	1		
Yes	15	(25.42)	1.64	(0.68–3.95)	
No	2	(40)	2.7	(0.38–19.01)	
Farm bed net condition					0.54
Does not stay at farms	15	(19.74)	1		
No bed net	2	(40)	2.7	(0.38–18.98)	
Bed net with holes	8	(22.22)	1.4	(0.5–3.96)	
Bed net without holes	7	(30.43)	2.02	(0.65–6.24)	
Goes to forest in the evening					0.015
No	15	(20)	1		
Yes	17	(26.15)	3.5	(1.27–9.61)	
Goes logging in the evening					0.002
No	28	(20.9)	1		
Yes	4	(66.67)	20.98	(3.1–141.94)	
Collects firewood in the evening					0.88
No	29	(22.83)	1		
Yes	3	(23.08)	1.11	(0.28–4.46)	
Goes hunting in the evening					0.82
No	25	(25.77)	1		
Yes	7	(16.28)	1.18	(0.28–5.06)	
Collects forest food in the evening					0.06
No	22	(20.18)	1		
Yes	10	(32.26)	2.55	(0.97–6.7)	
Goes fishing in the evening					0.07
No	27	(22.31)	1		
Yes	5	(26.32)	3.23	(0.9–11.59)	
MAPARES cohort participation					0.77
Partial	16	(18.82)	1		
Full	16	(29.09)	1.14	(0.47–2.76)

**Table 3:** Odds ratios for association between exposure variables and recent *Plasmodium vivax* exposure by serology, adjusted for age and sex only

Variable	n recently exposed (%)		OR	(95% CI)	p
Age	38.7	(16.33)	1.02	(0.99–1.04)	0.27
Sex					0.05
Male	7	(10.61)	1		
Female	17	(22.97)	2.6	(0.99–6.78)	
Drinks in hamlet in evening					0.93
No	14	(15.91)	1		
Yes	10	(19.23)	1.05	(0.39–2.82)	
Frequency of evening drinking in hamlet					0.4
Daily	4	(21.05)	1.71	(0.49–6.03)	
Less than daily	20	(16.67)	1		
Evening TV watching					0.16
Does not watch TV	12	(14.12)	1		
Watches TV at home	3	(12.5)	0.84	(0.21–3.41)	
Watches TV in another house	9	(29.03)	2.59	(0.91–7.38)	
Uses bed net in hamlet house					0.4
Never	2	(50)	1		
Sometimes	3	(10.71)	0.19	(0.02–2.13)	
Always	19	(18.27)	0.3	(0.04–2.45)	
Condition of bed net in hamlet house					0.18
No bed net	0	(0)			
Bed net with holes	7	(12.96)	1		
Bed net without holes	17	(20.73)	1.97	(0.73–5.34)	
Number of children in household	2.17	(1.52)	0.73	(0.55–0.96)	0.025
Household bed net ratio					0.84
1–2 people per net	8	(19.05)	1		
> 2 people per net	16	(17.2)	0.9	(0.35–2.36)	
Other *P. vivax* seropositive household member					0.9
No	16	(16.84)	1		
Yes	8	(17.78)	0.94	(0.36–2.44)	
Hamlet house structure					0.11
On ground	11	(23.91)	1		
On stilts	36	(10.53)	0.27	(0.09–0.81)	
Stays overnight at farm					0.32
No	11	(14.47)	1		
Yes	13	(20.31)	1.58	(0.64–3.92)	
Duration of stay at farm					0.55
Does not stay	11	(14.47)	1		
Stays up to 1 week	10	(20)	1.48	(0.57–3.89)	
Stays more than 1 week	3	(21.43)	2.06	(0.47–9.10)	
Drinks in evening at farm					0.61
Does not stay at farms	11	(14.47)	1		
Yes	5	(20.83)	1.61	(0.49–5.36)	
No	8	(20)	1.57	(0.56–4.39)	
Uses net at farm					0.09
Does not stay at farms	11	(14.47)	1		
Yes	10	(16.95)	1.27	(0.49–3.3)	
No	3	(60)	8.96	(1.27–63.27)	
Farm bed net condition					0.18
Does not stay at farms	11	(14.47)	1		
No bed net	3	(60)	8.96	(1.27–63.32)	
Bed net with holes	6	(16.67)	1.3	(0.43–3.95)	
Bed net without holes	4	(17.39)	1.22	(0.34–4.39)	
Goes to forest in the evening					0.61
No	10	(15.38)	1		
Yes	14	(18.67)	1.31	(0.47–3.6)	
Goes logging in the evening					0.11
No	22	(16.42)	1		
Yes	2	(33.33)	4.69	(0.72–30.63)	
Collects firewood in the evening					0.54
No	21	(16.54)	1		
Yes	3	(23.08)	1.55	(0.38–6.28)	
Goes hunting in the evening					0.68
No	20	(20.62)	1		
Yes	4	(9.3)	0.71	(0.14–3.53)	
Collects forest food in the evening					0.86
No	19	(17.43)	1		
Yes	5	(16.13)	0.91	(0.3–2.74)	
Goes fishing in the evening					0.21
No	20	(16.53)	1		
Yes	4	(21.05)	2.36	(0.62–8.95)	
MAPARES cohort participation					0.44
Partial	12	(14.12)	1		
Full	12	(21.82)	1.44	(0.57–3.65)	

Consumption of rice wine in the evenings in the hamlets was very common amongst men (84%) and common amongst women (44%), and 16% of men and 8% of women (12% overall) reported to drink every evening (Table 1). Rice wine was usually consumed outdoors in groups and had multiple functions, such as to aid sleep and soothe aches and pains after a day spent working in the fields, to facilitate social interactions *(“first we invite each other for drinking, then we start talking”*), transactionally (*“paying for favours in wine”*), and for a range of traditional ceremonies. Rice wine consumption delayed sleeping time; the median sleeping time was 8 pm if not drinking (IQR 7–9 pm), 9 pm after drinking (IQR 9–10 pm). Evening drinking hindered bed net use as, when asked generally, 88.5% of survey respondents reported to always sleep under a bed net, but only 65.4% reported to sleep under a bed net after drinking in the evening. There was a non-significant trend towards increased risk of *P. falciparum* exposure amongst daily drinkers in multivariable analyses (OR 2.15, 95% CI 0.71–6.51) (Table 4). However, when restricting analysis to individuals who never slept at the farms, daily drinking was their main risk factor for recent *P. falciparum* exposure (OR 9.52, 95% CI 1.93–46.87, p = 0.006) (Table 4).

**Table 4:** Multivariable risk factor analysis for recent *Plasmodium falciparum* exposure, in whole population and separately for farm and non-farm sleepers

Variable	Whole population			Farm sleepers only			Non-farm sleepers only		
	OR	(95% CI)	p	OR	(95% CI)	p	OR	(95% CI)	p
Age	1.07	(1.03–1.12)	0.001	1.08	(1.02–1.16)	0.013	1.09	(1.03–1.15)	0.002
Sex			0.003			0.003			0.21
Male	1			1			1		
Female	7.89	(2.01–30.9)		14.23	(2.40–84.27)		2.86	(0.52–14.95)	
Frequency of evening drinking in hamlet			0.18						0.006
Daily	2.15	(0.71–6.51)					9.51	(1.93–46.87)	
Less than daily	1						1		
Number of children in household	0.89	(0.61–1.3)	0.55						
Household bed net ratio			0.32						
1–2 people per net	1								
> 2 people per net	0.5	(0.13–1.95)							
Other *P. falciparum* seropositive household member			0.19						
No	1								
Yes	2.23	(0.67–7.49)							
Hamlet house structure									0.01
On ground							1		
On stilts							0.12	(0.03–0.60)	
Duration of stay at farm			0.2			0.07			
Does not stay	1								
Stays up to 1 week	0.93	(0.34–2.55)		1					
Stays more than 1 week	3.88	(0.78–19.32)		6.99	(0.84–58.42)				
Goes to forest in the evening			0.14						
No	1								
Yes	2.4	(0.75–7.63)							
Goes logging in the evening			0.006			< 0.001			
No	1			1					
Yes	10.85	(2.01–58.51)		96.26	(8.58–1079.44)				
Goes fishing in the evening			0.17			0.028			
No	1			1					
Yes	3.11	(0.63–15.43)		7.99	(1.25–50.85)				

Watching television/DVDs (hereafter ‘TV’) in the evenings was a common social activity. Most often, people watched TV together in a central house (26.5%), particularly in Tu Nak, many or all of whom sit outside as there is not enough space in the house. Only 17.5% watched TV at home (thus indoors), reflecting low TV ownership (Table 1). Watching TV in the evenings at another’s house, a proxy for outdoor TV watching, was associated with increased risk of recent *P. vivax* exposure (OR 6.29, 95% CI 1.49–26.58) (Table 5).

**Table 5:** Multivariable risk factor analysis for recent *Plasmodium vivax* exposure, in whole population and for non-farm sleepers

Variable	Whole population			Non-farm sleepers only		
	OR	(95% CI)	p	OR	(95% CI)	p
Age	1.03	(1–1.08)	0.04	1.01	(0.97–1.06)	0.57
Sex			0.07			0.53
Male	1			1		
Female	3.61	(0.88–14.66)		1.89	(0.26–13.54)	
Evening TV watching			0.04			
Does not watch TV	1					
Watches TV at home	0.61	0.09–4.01)				
Watches TV in another house	6.29	(1.49–26.58)				
Number of children in household	0.68	(0.48–0.98)	0.04	0.47	(0.29–0.76)	0.002
Household bed net ratio						0.10
1–2 people per net				1		
> 2 people per net				4.94	(0.75–32.62)	
Hamlet house structure			0.13			
On ground	1					
On stilts	0.35	(0.09–1.37)				
Uses net at farm			0.04			
Does not stay at farms	1					
Yes	1.34	(0.45–3.99)				
No	17.57	(1.91–160.80)				
Goes logging in the evening			0.02			
No	1					
Yes	5.45	(1.26–22.76)				

#### Forest farm and field-based activities

There were 72 (45%) survey respondents who slept overnight at their forest farms and fields. The longest duration of stay was mostly less than 1 week and was not associated with recent malaria, but 10% of overnight sleepers stayed for 1 week to more than 1 month continuously, which was associated with recent exposure to *P. falciparum* malaria amongst farm sleepers (adjusted OR 6.99, 95% CI 1.22–40.01, p = 0.029) (Table 4).

Over 90% of farm sleepers reported to sleep under a ITN or LLIN, and qualitative research suggested that bed net use at farms was consistently high owing to perceived high insect nuisance. Evening sleeping times at farms were earlier than in the hamlets (median sleep time 7 pm, IQR 6–8 pm). Not sleeping under a bed net at the farm was associated with recent *P. vivax* exposure (adjusted OR 17.57, 95% CI 1.91–160.80) (Table 5).

Evening drinking also occurs at the farms and fields (18.1% of men and 16.9% of women sometimes drink), but intermittently rather than regularly, and was not associated with *P. falciparum* or *P. vivax* exposure (Tables 2, 3).

#### Forest and river activities

46% of respondents reported spending evening hours in the forest, for collecting wild plant foods (24%), hunting (32.5%), collecting firewood (11.3%) and logging (3.8%) (Table 1). Different forest activities had different exposure patterns. Respondents reported to go hunting at night, but sleep at their plot hut or village house, whereas logging required multi-night stays in the forest. Logging was strongly associated with recent exposure, particularly for *P. falciparum* (OR 10.9, 95% CI 2.01–58.51, p = 0.006) but also for *P. vivax* (OR 5.45, 95% CI 1.26–22.76, p = 0.02). Insecticide-treated hammock nets were rarely taken to the forest (only two individuals in the survey).

Evening fishing was reported by 20 (12.50%) respondents, mostly men (75%) and mostly in Xe Xua hamlet where the river is located. Men went fishing in small groups of 4–6 people and returned late in the evening (median 10 pm, IQR 9 pm–12 am). Evening fishing was weakly associated with recent *P. falciparum* exposure amongst farm sleepers (OR 7.99, 95% CI 1.10–58.04, p = 0.04) (Table 4).

#### Demographic, household and other variables

Increasing age was associated with increased risk of recent exposure to *P. falciparum,* less so for *P. vivax*. Females were at higher risk of recent malaria exposure in univariable analyses, an effect that strengthened after adjusting for evening and outdoor activities, which were more prevalent in males.

Higher numbers of children living in a household was associated with decreased risk of *P. falciparum* and *P. vivax* exposure. Household bed net ratio or other seropositive members were not associated with *P. falciparum* or *P. vivax* exposure in adjusted analyses. Having a hamlet house built on stilts rather than ground level was not associated with *P. falciparum* or *P. vivax* exposure overall, but was protective against *P. falciparum* exposure amongst non-farm sleepers (OR 0.12, 95% CI 0.03–0.60, p = 0.01) (Table 4).

### Sensitivity analyses

Restricting the analyses to only individuals with complete serology data (n = 104) led to minor fluctuations in effect sizes for most variables, except the effect of logging for *P. falciparum* exposure (OR attenuated from 10.9 to 3.3), but this may be affected by having only three loggers in the model with complete serology data. Participation in screening surveys was not associated with recent malaria exposure (Tables 2, 3). Different definitions for seropositivity were assessed, including defining a cut-off based on number of standard deviations above the negative control, or by applying a mixture model [32, 33], which in general resulted in a larger proportion of the population considered ‘seropositive’ and smaller effect sizes in risk factor analyses.

## Discussion

This study used a mixed-methods study design to identify and further explain local risk factors for malaria infection in Central Vietnam. Evening outdoor activities such as drinking and TV watching were the main hamlet-based activities linked to recent *P. falciparum* and *P. vivax* exposure, respectively, but were not associated with malaria exposure amongst farm sleepers. Instead, farm sleepers, who spend variable proportions of time residing at their farms away from the hamlet throughout the year, were more likely to be recently exposed to *P. falciparum* malaria if they went fishing or logging in the evenings. At farms, TV watching was unavailable and drinking occurred much less frequently, whereas fishing and logging were more common amongst farm sleepers than non-farm sleepers. Apart from bed net use, no evening activities specifically conducted whilst staying overnight at forest farms and fields could be identified as risk factors.

The main findings of this study point to the importance of a micro-epidemiological approach in pre-elimination settings with shifting epidemiology. Broadly categorized behaviours previously recognized as risk factors, such as overnight sleeping at forest farms and bed net use in the village [4–6], were not associated with recent malaria exposure in this adult study population in a low prevalence setting. Instead, ongoing malaria transmission appears to be maintained by activities that delay or disrupt sleeping in a permanent structure in which a bed net could be hung, including evening drinking gatherings, fishing, logging in the forest and outdoor TV watching. Additionally, no bed net use at forest farms remained a risk factor for recent *P. vivax* exposure. Thus, this study provides detailed information on human behaviours that contribute to residual transmission, in contrast to previous literature in which residual transmission has been largely defined and characterized with respect to vector behaviour [34, 35] with comparatively limited consideration of human behaviours that maintain residual transmission (exceptions include [36, 37]).

Females were more likely to be classified as recently exposed by serology, and the effect of female sex became more pronounced after adjustment for behavioural-related risk factors, a finding that is in contrast to previous studies in this area that found males to be at higher risk of malaria when measured by PCR or microscopy rather than serology [4, 6, 38]. In the full MAPARES cohort, though numbers were small, descriptively an age-dependent sex difference in seropositivity was observed: there was no sex difference in seropositivity up to 10 years of age or after 50 years of age, but higher proportions of seropositive women aged 10–49. This might be explained by estrogen-mediated sex differences in immune response to parasitic infections, including *Plasmodium* parasites [39–43]. It is also possible that the analyses were confounded by unknown exposure-related behaviours that were more prevalent amongst females than males. Previous studies that have found higher seropositivity rates in females to have not distinguished intrinsic biological factors versus risk behaviours as determinants [23, 25, 44]. The association between sex and seropositivity is inconsistent in the literature (e.g., males at higher risk in [45], no difference in [46, 47]), and may vary by antibody [23, 25] as well as by study setting. More detailed exploration of sex differences in malaria seropositivity outcomes should be considered in future studies.

### Limitations

Though serological markers can be used to describe fine-scale heterogeneity in malaria infection in both high and low transmission settings [24, 27, 48, 49], it nonetheless remains difficult to define serological outcomes that accurately reflect recent malaria infection using a limited set of antibodies [50]. Specificity was favoured over sensitivity in selecting the algorithm used to generate the seropositivity cut-points, in order to define the primary outcome of ‘recent malaria exposure’. Alternative definitions of seropositivity were considered, but it was considered incongruous to use cut-offs that defined a larger proportion of the study population as recently exposed, given there were so few current infections detected during the cohort study period. Nonetheless, the specific definition used may have excluded some recently exposed individuals with attenuated antibody responses. The analyses for *P. falciparum* are considered more reliable than for *P. vivax* to detect risk factors for recent exposure to new infections as the latter could not be separated from recrudescence for *P. vivax*.

This study was limited by the sample size, which in the cross-sectional survey was not sufficiently powered for the various sub-group analyses, and complete serological data was only available for 104 of 160 (65%) respondents, reflecting incomplete participation in the cohort study wherein complete serological data was only available for 58.6% of respondents. The sampling frame was restricted to adults as it was anticipated that there would be a high non-response rate from children requiring parental consent to participate as many children reside at school during the week and then accompany their parents to the farms on the weekend. Nonetheless, this restricts the generalizability of findings from this study, including no association between hamlet bed net use and recent malaria exposure, to adult populations in this setting only. Furthermore, a complete net integrity assessment was not undertaken, which restricts the validity of the bed net condition variable. The proportion considered recently exposed in this analysis was higher than in the MAPARES cohort overall (10.4% for *P. falciparum* and 12.3% for *P. vivax*), largely reflecting the exclusion of children in the cross-sectional survey, who had lower malaria antibody levels (Kattenberg et al., pers. comm.).

Beyond these sample limitations, in general there are challenges in conducting studies in low prevalence settings, especially when risk activities are practiced by a small proportion of the population (e.g. logging in the forest) that limit statistical power to detect important risk factors and calculate meaningful confidence intervals. Triangulating quantitative against qualitative findings partly ameliorates this challenge, as in-depth interviews and observation of the setting provides additional context that can support or refute the quantitative results.

### Implications and further research

As residual transmission appears to be maintained by outdoor evening activities that disrupt or delay sleeping, long-lasting insecticidal hammock nets may be of limited utility for achieving further reductions in malaria transmission, despite being designed for similar forest malaria settings in Vietnam [51]. Declining malaria incidence in Tra Cang in recent years demonstrates that malaria can be very well controlled with existing interventions, which have combined LLIN distributions, ensuring access to diagnosis and treatment, and active case detection and treatment campaigns despite ongoing structural vulnerability to malaria. In addition to these malaria control activities, there may be a role for supplementary vector control tools such as personal topical repellents for use at farms and in the forest, or spatial repellents for evening gatherings in hamlets, though neither tool shows consistent efficacy [52, 53], and effectiveness is dependent on acceptance and sufficient use by the target population [54].

## Conclusions

Persistent malaria transmission in Tra Cang, Central Vietnam, appears to be maintained by outdoor evening activities that delay or disrupt sleeping in a permanent structure in which a bed net could be hung, including evening drinking gatherings, fishing, logging in the forest and outdoor TV watching, as well as non-use of bed nets at forest farms and fields. In addition to existing malaria control efforts, vector control tools adapted for use during outdoor evening activities in villages as well as at farms, forest and river locations should be incorporated into malaria elimination efforts in Central Vietnam. Effectively targeting residual malaria transmission remains an area of active research, to which studies using mixed-methods designs can contribute by identifying context-specific determinants of persisting malaria risk, which may guide further research and application of appropriate interventions in settings in which residual transmission occurs.

## Additional files

**Additional file 1.** Mean percent-positivity (PP) cut-points used to define seropositivity categories in 1st and 6th survey, and minimum changes in mean PPs between surveys used to define stable or increased antibody level.

**Additional file 2.** Seropositivity categories at first and sixth screening survey, by antibody.
