# Supplementary material for: Micro-epidemiology of malaria in an elimination setting in Central Vietnam
Source: Malar J. 2018 Mar 19;17:119. doi: 10.1186/s12936-018-2262-0 (PMC5859719; doi:10.1186/s12936-018-2262-0)
Supplement: Supplementary file 1 — Additional file 1. Mean percent-positivity (PP) cut-points used to define seropositivity categories in 1st and 6th survey, and minimum changes in mean PPs between surveys used to define stable or increased antibody level. [file 12936_2018_2262_MOESM1_ESM.docx]

**Additional File 1**

**Supplementary Table:** Mean percent-positivity (PP) cut-points used to define seropositivity categories in 1^st^ and 6th survey, and minimum changes in mean PPs between surveys used to define stable or increased antibody level.

|  | ***P. falciparum* antibodies**  PP (%) | | ***P. vivax antibodies***  PP (%) | | | |
| --- | --- | --- | --- | --- | --- | --- |
| Seropositivity category | Pf AMA1 | Pf GLURP2 | | Pv AMA1 | Pv MSP1 |  |
| Cat 4: Strong positive | 44.01 | 37.31 | | 37.33 | 15.33 |  |
| Cat 3: Weak positive | 15.66 | 16.72 | | 11.84 | 8.73 |  |
| Cat 2: Grey zone | 6.52 | 9.36 | | 3.91 | 4.67 |  |
| Cat 1: Negative | <6.52 | <9.36 | | <3.91 | <4.67 |  |
| Minimum relative PP decrease (Cat 3 & 4 only) | -12.8 | -12.8 | | -12.8 | -12.8 |  |
| Minimum absolute PP increase (Cat 1 & 2 only) | +1.8 | +1.4 | | +2.7 | +1.4 |  |
