# Supplementary material for: Micro-epidemiology of malaria in an elimination setting in Central Vietnam
Source: Malar J. 2018 Mar 19;17:119. doi: 10.1186/s12936-018-2262-0 (PMC5859719; doi:10.1186/s12936-018-2262-0)
Supplement: Supplementary file 2 — Additional file 2. Seropositivity categories at first and sixth screening survey, by antibody. [file 12936_2018_2262_MOESM2_ESM.pptx]

## Slide 1
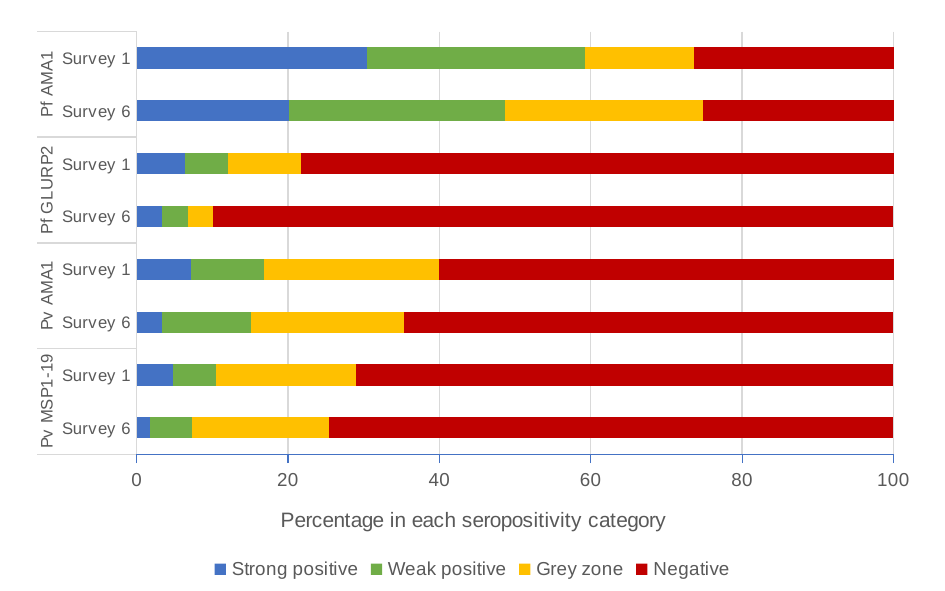

### Chart
| Category | Strong positive | Weak positive | Grey zone | Negative |
|---|---|---|---|---|
| Survey 6 | 1.818181818181818 | 5.454545454545435 | 18.18181818181818 | 74.54545454545448 |
| Survey 1 | 4.838709677419355 | 5.64516129032258 | 18.54838709677419 | 70.96774193548345 |
| Survey 6 | 3.361344537815126 | 11.76470588235294 | 20.16806722689076 | 64.70588235294024 |
| Survey 1 | 7.199999999999997 | 9.6 | 23.2 | 60.0 |
| Survey 6 | 3.361344537815126 | 3.361344537815126 | 3.361344537815126 | 89.91596638655435 |
| Survey 1 | 6.451612903225806 | 5.64516129032258 | 9.67741935483871 | 78.22580645161288 |
| Survey 6 | 20.16806722689076 | 28.57142857142857 | 26.05042016806723 | 25.21008403361344 |
| Survey 1 | 30.4 | 28.8 | 14.4 | 26.4 |
